# De-implementation of urodynamics in The Netherlands after the VALUE/VUSIS-2 results: a nationwide survey

**DOI:** 10.1007/s00192-018-3648-9

**Published:** 2018-04-20

**Authors:** Bianca B. Mengerink, Willianne L. D. M. Nelen, Sanne A. L. van Leijsen, John P. F. A. Heesakkers, Kirsten B. Kluivers

**Affiliations:** 1 Department of Obstetrics & Gynecology, Radboud University Nijmegen Medical Center, 791, Postbus 9101, 6500 HB Nijmegen, The Netherlands; 2 Department of Urology, Radboud University Nijmegen Medical Center, Nijmegen, The Netherlands; 3 Department of Obstetrics & Gynaecology, Jeroen Bosch Hospital, Den Bosch, The Netherlands

**Keywords:** De-implementation, Midurethral sling surgery, Stress urinary incontinence, Urodynamics

## Abstract

**Introduction and hypothesis:**

We aimed to estimate the level of de-implementation of preoperative routine urodynamics (UDS) before stress urinary incontinence (SUI) surgery in The Netherlands and to analyze facilitators and barriers. Routine UDS was performed by 37% of the medical specialists in 2010. We hypothesized that the recommendations from the recent Value of Urodynamics prior to Stress Incontinence Surgery (VUSIS) and Value of Urodynamic Evaluation (ValUE) studies would have been followed by a reduction of routine UDS.

**Methods:**

A national survey was performed among all Dutch gynecologists and urologists dealing with SUI in daily practice. The questionnaire contained two parts: (1) respondents’ characteristics and their actual care concerning preoperative UDS, and (2) facilitators and barriers.

**Results:**

The response rate was 41% (127/308). Of the respondents, 93% (*n* = 118) did not perform routine UDS in the preoperative workup for women in this group. Professional characteristics associated with not following the recommendations were profession urologist, academic hospital, and a lower number of midurethral sling (MUS) placed yearly. Facilitators to follow the recommendation not to perform routine UDS were adequate design of the VUSIS-II study and outcome and recommendations from the studies. Barriers not to follow the recommendation were believe in the additional value of UDS, especially the pressure transmission ratio, and the presence of detrusor overactivity.

**Conclusion:**

According to respondents to this questionnaire, VUSIS-II and ValUE study results are well implemented in The Netherlands. The vast majority of respondents replied as not performing routine preoperative UDS in women with primary, uncomplicated (predominant) SUI. Therefore, there is no need for a further de-implementation strategy.

## Introduction

Urodynamics (UDS) is an attempt to enhance the understanding of lower urinary tract function and reveal the underlying pathophysiology responsible for the patient’s complaints. Nevertheless, the evidence that UDS contribute to the final outcome of treatment is limited [1], and disadvantages of the procedure, such as patient discomfort urinary tract infection (UTI) risk, and costs, are known.To provide evidence, recently, two large randomized controlled trials (RCTs) studied the value of UDS in women with uncomplicated (predominant) stress urinary incontinence (SUI) who were eligible for SUI surgery [2, 3]. Recommendations based on the outcomes of both trials were to renounce from routine preoperative urodynamic testing in these patients. Both the Value of Urodynamics prior to Stress Incontinence Surgery (VUSIS-II) and Value of Urodynamic Evaluation (ValUE) trials were designed as multicenter noninferiority RCTs in women who did not undergo a prior operation for SUI, who had previously failed conservative therapy, and who were candidates suited for surgical therapy. The VUSIS-II trial was conducted in The Netherlands by our study consortium in a group of female patients who all received UDS prior to surgery. When UDS were discordant with clinical assessment, these women were randomized to either immediate midurethral sling (MUS) surgery or an individually tailored treatment based on urodynamic findings. The conclusion of the VUSIS trial was that MUS surgery in uncomplicated SUI without UDS was not inferior to the individually tailored treatment based on urodynamic findings [2]. The American ValUE trial randomized between an office evaluation without UDS versus UDS in addition to the office evaluation before the planned surgery. The ValUE study also concluded that preoperative office evaluation was not inferior to evaluation with additional urodynamic testing after 1 year [3].

RCTs are the most rigorous way of determining whether a cause–effect relation exists between therapy and outcome. Ultimately, the reason for conducting trials is to deliver evidence. For practice change, doctors should be aware of results of RCT trials and clinical practice guidelines. Regarding this care in SUI, the Dutch national guideline on urinary incontinence (UI) for hospitals was rewritten and published in May 2014 [4]. The recommendations on routine UDS in women before conservative treatment were: do not routinely perform UDS for patients who are treated conservatively (level B recommendation). Recommendations on preoperative UDS in women with uncomplicated SUI seeking therapy were based on the ValUE trial [3] outcomes only, because the VUSIS-II trial results were not yet published. Before invasive treatment the recommendation has not been changed: to perform UDS in case test results would change the choice of treatment (level C recommendation).

However, until now, it was unknown whether the publication of the RCTs and the revised guideline had changed the current clinical practice. In 2010, before the outcome of VUSIS and ValUE, a survey was conducted in The Netherlands to determine the use of UDS at that time by professionals [5]. According to gynecologists and urologists (*n* = 163), 37% replied that their common policy was to always perform preoperative UDS in women with (predominant) SUI, 48% performed UDS on indication, and 15% never performed UDS in this group of women.

In the survey reported here, we evaluated the actual position of routine preoperative UDS in The Netherlands. The objectives were to determine how many professionals still routinely perform preoperative UDS in women with primary uncomplicated (predominant) SUI and to evaluate what determinants influence the use of UDS. Secondary we explored facilitators and barriers to further implement VUSIS-II and ValUE trial results in case further de-implementation strategies were needed. We hypothesized that daily practice had changed toward fewer professionals performing routine UDS in such women who opt to undergo SUI surgery.

## Materials and methods

### Study design

We conducted a cross-sectional survey in The Netherlands using an online questionnaire. This survey was performed among all gynecologists and urologist in The Netherlands who manage women with SUI in daily practice. No ethical review board approval was needed.

### Questionnaire

The questionnaire consisted of two parts. The first part contained 20 multiple choice questions on respondent characteristics, the setting in which the care provider works, and current care practice after publication of the VUSIS-II and ValUE trials. Examples of characteristics were age, gender, and type of hospital in which the professional worked. Respondents were asked what their actual care was concerning preoperative UDS for women with primary uncomplicated SUI with (predominant) SUI symptoms who had previously failed conservative therapy and were candidates for surgical therapy.

The second part consisted of 45 Likert scale items concerning facilitators and barriers on the preoperative routine evaluation with UDS. The five-point Likert scale items ranged from 1 = complete disagreement to 5 = complete agreement. There was a comment box at the end of the questionnaire for participants to clarify their answers or give additional facilitators or barriers. A summary of the Checklist for Reporting Results of Internet E-Surveys (CHERRIES) [6] is given in Table 5 in the “Appendix”.

#### Barriers and facilitators analysis

We used two theoretical models to identify influencing factors: facilitators and barriers [7, 8]. These models consisted of four domains: characteristics of the innovation itself (e.g., outcome of VUSIS trial), professionals’ characteristics (e.g., urologist versus gynecologist), patients’ characteristics (e.g., symptoms and signs), and characteristics of the context in which the innovation is applied (e.g., legislation). To identify specific facilitators and barriers, we started with a qualitative study and narrowed toward a questionnaire. We selected seven professionals representing our questionnaire target population (urologists and gynecologist, academic and nonacademic) and performed semistructured interviews. The structure of all interviews was identical: we started with explorative open questions to identify possible factors related to their reasons for either performing or not performing UDS prior to SUI surgery. Subsequently, we asked questions about all factors potentially related to routinely performing UDS, suggested by the models. The interviews took about 15 min and all were audio taped and transcribed. We identified factors and placed them in the appropriate domain (“Appendix” Table 6). If factors were suggested as being both a facilitator and a barrier, we denominated them as both facilitator and barrier. Factors were finaly stated in the second part of the questionnaire with a Likert scale (see “Appendix C” for the whole questionnaire). Clinical cases were presented, and questions were asked on the preferred workup. There was an open question on possible de-implementation strategies.

### Study population

We selected all gynecologists registered in the Dutch Pelvic Floor Society, a subdivision of the Dutch Society of Obstetrics and Gynecology (NVOG), and all urologists working in the field of functional and reconstructive urology and registered at the Dutch Urological Association (NVU). The number of target specialists was 238 gynecologists and 70 urologists based on information of the professional societies. Databases of both societies were used to obtain contact details of the study population.

After development and pilot testing of the questionnaire, we approached all target medical specialists by email in June 2015 and invited them to voluntary fill in a web-based questionnaire. We additionally sent two reminders in a 2-month period to those who did not or only partly responded. The online questionnaire system did not accept unanswered items, and respondents who quit the questionnaire before completing it were excluded.

### Statistical analysis

Facilitators and barriers were answered on a 5-point Likert scale; a score 4 (agree) and 5 (completely agree) were defined as agreement on these statements. Those facilitators and barriers that correlated as being statistically significant with professionals performing routine UDS or no routine UDS are listed. Data were analyzed by using Statistical Package for the Social Sciences (SPSS) version 22. We used descriptive statistics. Categorical variables were compared using the Fisher’s exact test. A *p* value of <0.05 was considered statistically significant.

## Results

**Fig. 1:**
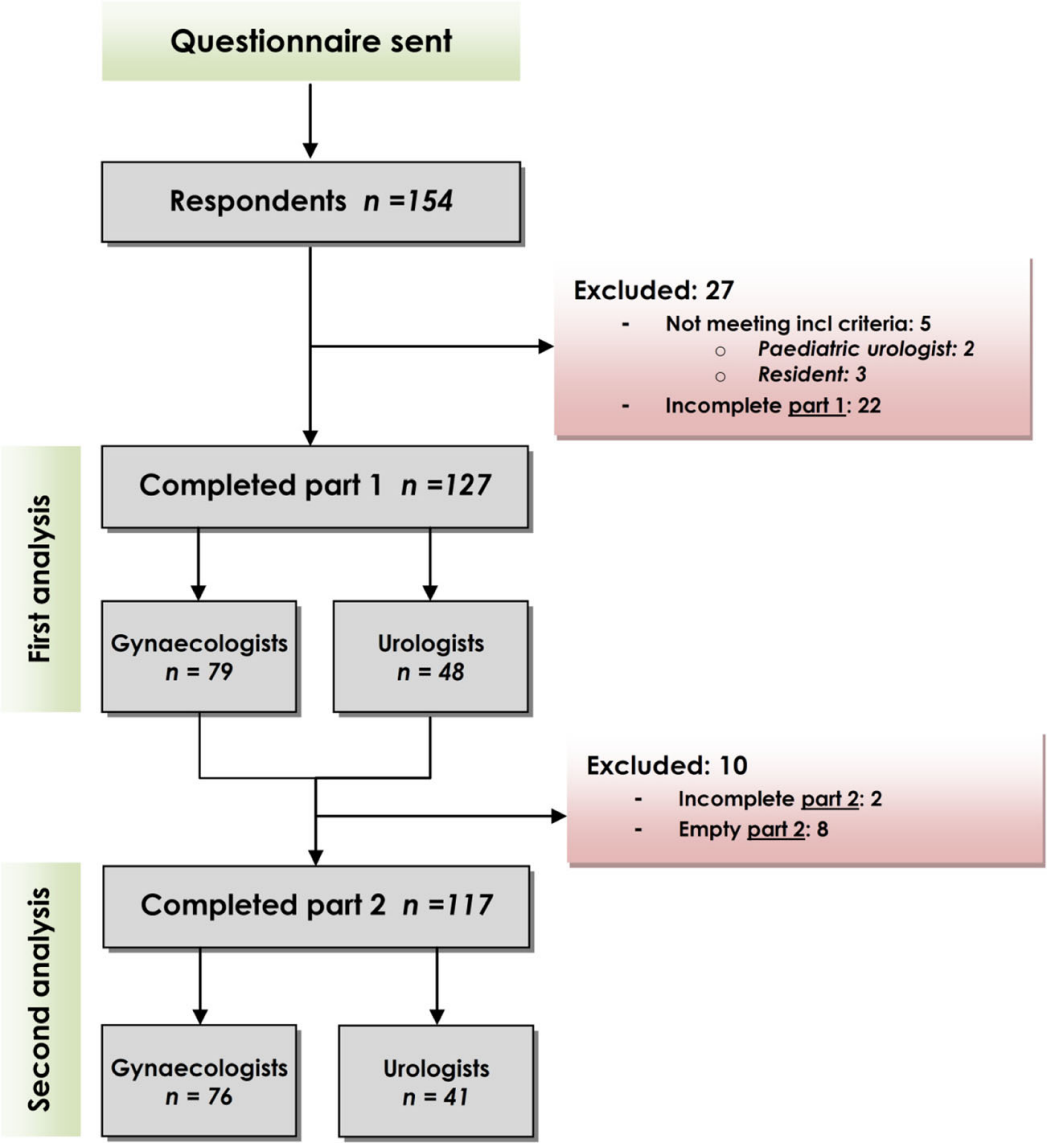
Responding participants

The survey was conducted from June until August 2015. We received 127 complete responses for analysis, and 27 questionnaires of 154 responses were excluded (see Fig. 1 for excluded respondents). Response rates for the target specialists were among gynecologists 33% (79/238) and among urologists 69% (48/70), resulting in a total response rate of 41%. The respondents’ characteristics are shown in Table 1.

**Table 1:** Characteristics of participating professionals (*n* = 127)

Characteristics		Total* n* (%)	Gynecologist* n* (%)	Urologist* n* (%)
Number of professionals		127	79	48
Gender	Male	65 (51%)	31 (39%)	34 (71%)
	Female	62 (49%)	48 (61%)	14 (29%)
Years of experience	<5 years 5–10 years 10–20 years >20 years	26 (20%) 32 (25%) 43 (34%) 26 (20%)	17 (22%) 22 (28%) 24 (30%) 16 (20%)	9 (19%) 10 (21%) 19 (40%) 10 (21%)
Type of hospital	University Teaching Nonteaching	18 (14%) 64 (50%) 45 (35%)	10 (13%) 42 (53%) 27 (34%)	8 (17%) 22 (46%) 18 (38%)
Area of specialization	Urogynecology 20 (16%) All-round gynecology 79 (44%) Functional and reconstructive urology 26 (16%) Allround urology 22 (17%)			
MUS yearly in own hospital	0–10 10–50 50–100 >100	4 (3%) 74 (58%) 41 (32%) 8 (6%)	0 (0%) 49 (62%) 25 (32%) 5 (6%)	4 (8%) 25 (52%) 16 (33%) 3 (6%)
Operations yearly by specialist	0–10 10–50 >50	27 (21%) 93 (73%) 7 (6%)	10 (13%) 67 (84%) 2 (3%)	17 (35%) 26 (54%) 5 (10%)
Type of procedure^a^	Retropubic sling Transobturator sling Minisling	12^b^ (SD 20) 27^b^ (SD 28) 7^b^ (SD 19)	11^b^ (SD19) 26^b^ (SD 23) 8^b^ (SD 22)	12^b^ (SD 13) 28^b^ (SD 34) 5^b^ (SD 12)
Able to interpret UDS	Yes No	111 (87%) 16 (13%)	64 (81%) 15 (19%)	47 (98%) 1 (2%)

*MUS* midurethral sling,* UDS* urodynamics,* SD* standard deviation

^a^Respondents were asked how many times a year these techniques were performed in their clinic for women with a first episode of (predominant) stress urinary incontinence

^b^Mean numbers for each procedure

Ninety-three percent (93%, *n* = 118) of the respondents replied that they do not perform routine UDS in the preoperative workup of women with primary uncomplicated (predominant) SUI. (Table 2).

**Table 2:** Reported actual care and changes since publication of Value of Urodynamics prior to Stress Incontinence Surgery and Value of Urodynamic Evaluation(VUSIS-II/VALUE) trial results

Care/changes in care		Total* n* (%)	Gynecologist* n* (%)	Urologist* n* (%)
Number of professionals		127	79	48
Changes since VUSIS and ValUE ^a^	Yes	77 (61%)	58 (73%)	19 (40%)
	No	50 (39%)	21 (27%)	29 (60%)
Actual care ^b^	Routine UDS	9 (7%)	2 (3%)	7 (15%)
	No routine UDS	118 (93%)	77 (97%)	41 (85%)

*UDS* urodynamics

^a^Respondents were asked if a change was seen regarding the preoperative UDS since VUSIS-II and ValUE study results were published

^b^Respondents were asked what their actual care was regarding performing preoperative UDS

Sixty-one percent (*n* = 77) experienced a change in their clinic in performing preoperative UDS since the publication of VUSIS-II and ValUE trial results, where a difference was seen for gynecologists compared with urologists (73% vs. 40%, respectively, experienced change; *p * < 0.01). Determinants associated with (or not) routinely performing UDS are shown in Table 3. We found a correlation with the type of profession, where gynecologists performed less routine UDS than urologists. Also, a correlation was found between type of hospital, with no routine UDS performed in nonteaching hospitals at all. The number of MUS placed yearly correlated with the outcome: in clinics where UDS was not routinely performed, more MUS were placed yearly. Working in an inclusion center of the VUSIS-II study and knowledge of the VUSIS-II and ValUE trial outcomes were not associated with routinely performing UDS. Specialists estimated themselves with percentages of performing routine UDS before and after the publication of the RCTs; means of these percentages are shown in the bottom two rows of the table, sorted by the two groups of routine versus no routine UDS.

**Table 3:** Relationship of professional and hospital determinants on their actual mode of care concerning not routinely or routinely performing preoperative urodynamics (UDS) for women with (predominant) stress urinary incontinence (SUI)

Characteristics of professionals		Routine UDS* n* (%)	No routine UDS* n* (%)	*P* value
Number of professionals		9	118	
Gender	Male	7 (78%)	58 (49%)	ns
	Female	2 (22%)	60 (51%)	
Profession	Gynecologist	2 (22%)	77 (65%)	0.03
	Urologist	7 (78%)	41 (35%)	
Years of experience	<5 years	2 (22%)	24 (20%)	ns
	5–10 years	2 (22%)	30 (26%)	
	10–20 years	4 (44%)	39 (33%)	
	>20 years	1 (11%)	25 (21%)	
Type of hospital	Academic	5 (56%)	13 (11%)	<0.01
	Teaching	4 (44%)	60 (51%)	
	Nonteaching	0 (0%)	45 (38%)	
MUS yearly in own hospital	0–10	3 (33%)	1 (1%)	<0.01
	10–50	4 (44%)	70 (59%)	
	50–100	2 (22%)	39 (33%)	
	>100	0 (0%)	8 (7%)	
Performing MUS myself	Yes	6 (67%)	103 (87%)	ns
	No	3 (33%)	15 (13%)	
Able to interpret UDS	Yes No	9 (100%) 0 (0%)	102 (86%) 16 (14%)	ns
UDS performed by (multiple answers allowed)	Me or colleague Physician assistant Nurse Referral to other division	3 (33%) 1 (11%) 6 (67%) 0 (0%)	9 (8%) 20 (17%) 61 (52%) 36 (31%)	ns
Working in inclusion centre	Yes No	3 (33%) 6 (67%)	36 (31%) 82 (69%)	ns
Changes since VUSIS-II/ValUE publication	Yes] No	1 (11%) 8 (89%)	76 (64%) 42 (36%)	<0.01
knowledge of VUSIS-II study outcome	No Neutral Yes	2 (22%) 1 (11%) 6 (67%)	15 (13%) 16 (14%) 87 (74%)	ns
knowledge of ValUE study outcome	No Neutral Yes	4 (44%) 0 (0%) 5 (56%)	27 (23%) 31 (26%) 60 (51%)	ns
Performing UDS before VUSIS-II/ValUE publication	Mean percentage of women	90%	36%	
Performing UDS after VUSIS-II/ValUE publication	Mean percentage of women	83%	15%	

*VUISS-II* Value of Urodynamics prior to Stress Incontinence Surgery,* VaLUE* Value of Urodynamic Evaluation,* MUS* midurethral sling, * ns* not significant

*P* values measured by Fisher’s exact test

All significant facilitators of not routinely performing UDS and barriers to routinely perform UDS are listed in Table 4. All nonsignificant facilitators and barriers are listed in “Appendix C”. Contributors to follow VUSIS-II/ValUE were the VUSIS-II study design, outcomes, and recommendations from these trials. Furthermore, professionals mentioned that voiding diary, uroflow/postvoid residual volume and physical examination gave them sufficient information, and they think the additional value of UDS is unclear. Barriers to de-implement routine performance of UDS were the opinion that it was of additional value, especially pressure transmission ratio and detrusor overactivity. They believed in the importance of UDS and satisfaction with the current logistic patient flow were contributing barriers to de-implementing the performance of routine UDS.

**Table 4:** Facilitators related to not routinely performing urodynamics (UDS) and barriers related to routinely performing UDS in the preoperative phase for women with stress urinary incontinene (SUI) according to professionals

Respondents	All (%) *n* = 117	No routine UDS (%) *n* = 109	Routine UDS (%) *n* = 8	*P* value
Facilitators				
Related to care provider				
I like the design of the VUSIS study	56% (66/107)	61% 66/109	0% (0/8)	<0.01
The combination of voiding diary, uroflow/postvoid residual volume, and physical examination gives me enough information	77% (90/107)	82% 89/109	13% (1/8)	<0.01
Related to study outcome				
Outcome of the VUSIS study	62% (73/107)	66% (72/109)	13% (1/8)	<0.01
Recommendation of the study VUSIS not to routinely perform UDS	65% (76/107)	69% (75/109)	13% (1/8)	<0.01
Uncertainty about the value of UDS	48% (56/107)	51% (56/109)	0% (0/8)	<0.01
Related to environmental factors				
The latest national guideline regarding urinary incontinence	67% (78/107)	71% 77/109	13% 1/8	<0.01
Barriers				
Reated to care provider				
I think the importance of urodynamics are wide	6% (8/107)	4% 4/109	50% 4/8	<0.01
UDS are additional value to me to know if there is detrusor overactivity	53% (61/107)	49% 53/109	100% (8/8)	<0.01
UDS are additional value to me to know the pressure transmission ratio	18% (18/107)	16% 17/109	50% (4/8)	0.01
Related to environmental factors				
The flow of patients, including the routinely performed urodynamics, was optimally regulated	5% (6/107)	2% 2/109	50% 4/8	<0.01

Value of Urodynamics prior to Stress Incontinence Surgery, Value of Urodynamic Evaluation

*P* values are measured with Fisher’s exact test

We asked professionals to estimate the percentage of women who received preoperative UDS for the indication of (predominant) SUI. Gynecologists reported 44% of women before publication of the VUSIS-II and ValUE trials and 14% at the time of this questionnaire, implying 30% fewer routine UDS. For urologists, the percentage before publication of study results was 41% and at the time of this questionnaire 31%, making an estimation of 10% fewer routine UDS. In case patients also suffered from a neurologic problem, 78% of respondents (*n* = 91) said they would do preoperative UDS. In patients who did not fulfill the “ordinary” characteristics (e.g., nulliparous or younger women), 49% of respondents reported they would perform preoperative UDS. In case the predominance of SUI was not clear, 62% of respondents reported they would perform preoperative UDS. Also, for patients with a large postvoid residual, a poor flow, or doubts regarding the reason of incontinence based on physical examination (e.g., urethra mobility), 55% of respondents said they would do preoperative UDS. Regarding possible de-implementation strategies, study participants suggested a brief summary or pocket information on VUSIS-II and ValUE results and recommendation and integration of the results in the national multidisciplinary guideline.

Results of the qualitative part of our study, concerning all mentioned possible facilitators and barriers, are listed in “Appendix” Table 6.

## Discussion

We performed a nationwide questionnaire study to evaluate whether preoperative UDS is still routinely performed in women with primary uncomplicated (predominant) SUI. Our self-reported data showed that Dutch gynecologists and urologists do not routinely perform UDS in this patient group (93%). There was a 34% decrease in UDS performance reported between 2010 and 2015. VUSIS-II and ValUE trial results are thus well implemented in The Netherlands. This is consistent with our hypothesis of a reduction of routine UDS.

The results of a survey in 2010 to assess the use of routine preoperative UDS in women with SUI in The Netherlands [5] showed greater use of UDS (34%) when compared with the actual care at the time of this survey. Our results also showed that professionals experienced a change of actual care since the publication of VUSIS-II and ValUE trial results, which is despite the national multidisciplinary guideline not yet having changed the recommendation. No other factors concerning UDS in previous years had changed (e.g., insurances, legislation), which makes a correlation to the change in actual care unlikely.

This study evaluated the de-implementation, or abandonment, of a specific investigative test prior to treatment. Abandoning ineffective medical practices and mitigating the risks of untested practices are important for improving patients’ health and containing healthcare costs. It is, furthermore, known that de-implementation might even be more difficult than implementation [9]. When large, well-done RCTs have contradicted the current medical practice, de-implementation seems logical, but it may meet fierce tactical resistance. Nevertheless, with this study, we have shown that de-implementation may occur through new evidence from multicenter RCTs without a specific de-implementation strategy.

Results showed that higher volume centers were correlated with fewer routine UDS tests. Specialists with more exposure may feel more comfortable with their preoperative workup compared with specialists with less exposure, whereas academic and teaching hospitals, on the contrary, do more routine UDS. The latter may be a result of a mix with more complex patients in a tertiary or referral hospital, where women with primary uncomplicated SUI will be treated in a more difficult context. Also, we found that more routine UDS were done by urologists than by gynecologists. An explanation might be that urologists do UDS more often in their own department and do not need to refer to somewhere else. In our cohort, none of the urologists replied that they refer patients to another department, compared with 44% of gynecologist. Furthermore, urologists were more able to analyze the UDS themselves (98%) versus gynecologists (81%). Cost effectiveness was neither a facilitator nor a barrier, according to respondents.

The ValUE study measured cost effectiveness in their study. For women with uncomplicated SUI and a confirmatory preoperative basic office evaluation, tens of millions of dollars could be saved annually in the United States by not performing urodynamic testing[10]. In the management of these women, eliminating this preoperative test has a major economic benefit. It might be reasonable to believe that, despite the differences with the Dutch healthcare system, this cost-effectiveness benefit also exists in our Dutch system. Participation in multicenter clinical trials is associated with better knowledge of the trial’s results, with a slightly better implementation of study results [11]. Nevertheless, in our study this determinant was not associated with professionals following study outcome recommendations.

Strengths of this study were the mixed methodology: we started with a qualitative study and narrowed toward a questionnaire. This offered a reliable representation of the attitudes about UDS and actual care in The Netherlands. The study represents the Dutch group of professionals who have SUI treatment as focus in daily practice, since urologists, gynecologists, and all types of hospitals are represented. This survey was conducted ~2 years after publishing of the VUSIS-II and ValUE study data, allowing a realistic timeframe for de-implementation. It would give professionals time to become familiar with study results by reading or hearing outcomes and recommendations, and to adjust to new developments and changes in current working strategies.

Some limitations of this study were that response rates, were moderate: 33% and 69% for gynecologists and urologists, respectively, and might be a point of criticism due to potential bias. However, this represented 41% of all Dutch professionals who see women with SUI in daily practice. To increase response rates, we used strategies advised by the Cochrane review for electronic questionnaires (e.g., white background, adding a picture, not mentioning “survey” in the e-mail subject line). Some of these strategies were impossible to follow [12]. There theoretically could have been a reporting bias favoring those who follow VUSIS-II and ValUE recommendations; we have no convincing evidence for this.

Attaining an accurate report on actual care by simply collecting numbers of UDS peformed using a self-reported professional questionnaire is not the most objective route, because the correlation between self-ratings of skill and actual performance in many domains is moderate to meager among health professionals [13]. It is likely that respondents provide socially desirable answers, resulting in a social desirability bias [14]. It is also known that self-reported adherence rates exceed objective rates, resulting in a median overestimation of adherence of 27% in a study on guideline adherence [15]. Nevertheless, with our study, we showed that professionals self-reported that they perform fewer UDS. 

To evaluate actual care in The Netherlands, patient record file research on performing preoperative UDS in women receiving MUS surgery would be usefull. The latter was recently done by an North American research group. They found that the use of UDS decreased following publication of the ValUE study—from 70% of all patients undergoing UDS prior to primary MUS in 2008–2009, versus only 41% in the contemporary cohort from 2014 to 2016 [16]. Lippman et al. conducted a study to evaluate whether practice patterns changed following publication of the ValUE trial. They found that in southern California, significantly fewer UDS are being performed regarding collected electronic medical record data over two timeframes. They found a statistically significant decrease from 39% of uncomplicated SUI patients undergoing UDS prior to sling surgery in a pre-VALUE period versus 20% in a post-VALUE period [17].

One of the de-implementation strategies suggested by the respondents was integrating the study results in The Nathional guideline. We suggest this as well. Despite our finding of adequate de-implementation in The Netherlands, ValUE and VUSIS-II results should be represented in the next version is of the national guideline for urinary incontinence as level A evidence.

## Conclusion

Results of the VUSIS-II and ValUE studies are widely implemented in The Netherlands. According to the responding gynecologists and urologists, UDS are not routinely performed in women with primary (predominant) SUI. A specific de-implementation strategy is therefore not necessary.

## Figures and Tables

**Table 5:**
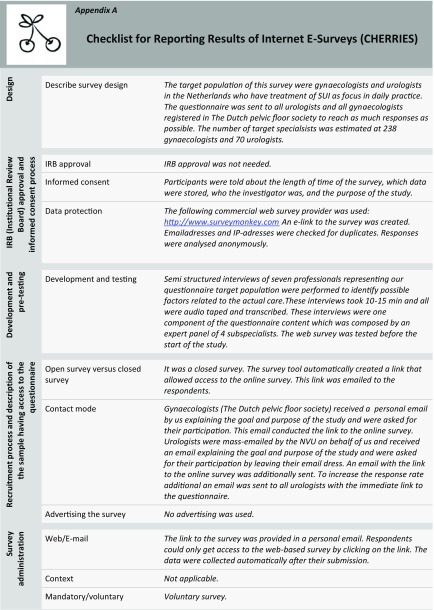

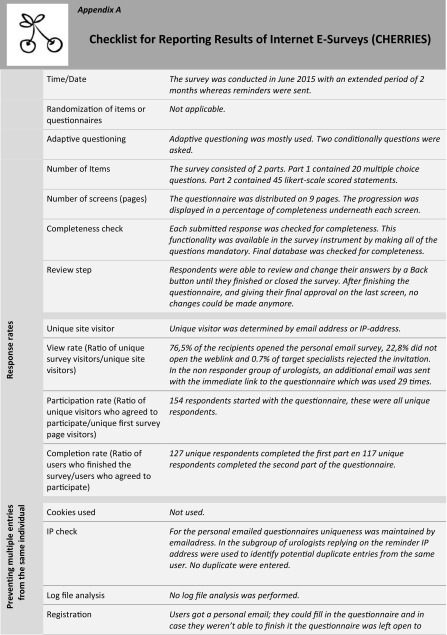

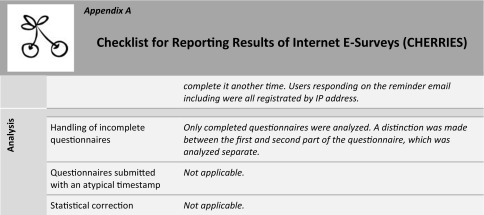
Checklist for Reporting Results of Internet E-Surveys (CHERRIES)

## Appendix B

**Table 6:** All potential facilitators and barriers possibly related to performing routine urodynamics (UDS) in the preoperative phase for women with stress urinary incontinence (SUI) destilated by semistructured interviews

Domain 1 Characteristics of *VUSIS-II and ValUE study results*	Domain 2 Professional characteristics	Domain 3 Patient characteristics	Domain 4 Context characteristics	Domain 5 Urodynamic investigation characteristics
Outcome of the VUSIS study (F) Recommendation of the VUSIS not to routinely perform UDS (F) I (do not) like the design of the VUSIS study (F/B)	The combination of voiding diary, uroflow/ postvoid residual volume and physical examination gives me enough information (F) I do not trust on UDS (F) If there might be an OAB component I’ll first treat with anticholinergics and do not need UDS (F) I think the importance of UDS are wide (B) UDS gives me evidence of stress incontinence (F) I think UDS are a forced examination (F) USD seem an unpleasant examination to me (F) I think performing UDS can be harmful (e.g., getting an UTI) I am used to perform urodynamics all my carrier (B) For counseling the patient regarding postoperative expectations I use urodynamic findings (B) I have to perform urodynamics myself (F)	I cannot apply these study results in daily practice because I treat another patient population (B) Patients feel UDS as stressful (F) Elderly patients (B) Neurological illness (B) Not belonging to “normal” SUI group (nulliparous, young women) (B) Unreliable patients in history (B) Predominance of SUI is less pronounced (B) Patients who not fill in/able to fill in their voiding diary (B) Unreliable voiding diary (B) Physical examination gives doubts to cause of incontinence (B) Large residue or poor flow (B)	The latest national guideline regarding urinary incontinence (F/B) Logistics without routinely UDS are faster (F) Logistics with routinely UDS are optimally regulated (B) It’s easier not to perform urodynamics (F) Performing UDS provides more work (F) There is less pressure on the equipment when you perform less UDS (F) To be stronger in claims I find evidence obtained by UDS usefull (B) promotes the cooperation between gynecologists and urologists (B) Cost effectiveness (F/B)	UDS are additional value to me to know if there is detrusor overactivity or reduced transmission or intrinsic sphincter deficiency (B) Uncertainty about the value or way of performing UDS (F) No demonstrable stress incontinence on UDS helps me in the decision to perform surgery (B) A low MUCP or low LPP on UDS could lead to a different treatment or a referral to a colleague (B)

*VUSIS-II and ValUE* Value of Urodynamics prior to Stress Incontinence Surgery,* ValUE* Value of Urodynamic Evaluation,* F* facilitator,* B* barrier,* UTI* urinary tract infection,* MUCP* maximal urethral closure pressure,* LPP* leak-point pressure,* OAB* overactive bladder
